# Associations of tongue and hyoid position, tongue volume, and pharyngeal airway dimensions with various dentoskeletal growth patterns

**DOI:** 10.1371/journal.pone.0326092

**Published:** 2025-06-13

**Authors:** Jin Hye Kang, Jeong-Yun Kim, Nayansi Jha, Seok-Ki Jung, You-Sun Lee, Yoon-Ji Kim

**Affiliations:** 1 Department of Orthodontics, Korea University Graduate School of Medicine, Seoul, Korea; 2 Department of Orthodontics, Korea University Graduate School of Clinical Dentistry, Seoul, Korea; 3 Department of Orthodontics, Asan Medical Center, University of Ulsan College of Medicine, Seoul Korea; 4 Department of Orthodontics, Korea University Guro Hospital, Seoul, Korea; 5 Department of Orthodontics, Korea University Anam Hospital, Seoul, Korea; Universidade Federal Fluminense, BRAZIL

## Abstract

**Objective:**

This study investigated the association between tongue and hyoid position, tongue volume, and pharyngeal airway dimensions with craniofacial growth patterns in the sagittal, vertical, and transverse planes.

**Methods:**

Cone beam computed tomography was used to assess 185 non-growing subjects (mean age, 28.7 ± 9.5 years). Multivariate linear regression analyses evaluated relationships between tongue and airway variables, and cephalometric/dental arch measurements.

**Results:**

Class III skeletal patterns—reflected by lower ANB and higher APDI—were significantly correlated with anteriorly positioned hyoids (ANB: β = 0.249; APDI: β = –0.291), and lower tongue positions at the tongue tip (ANB: β = –0.231; APDI: β = 0.166) and in the posterior area (ANB: β = –0.186; APDI: β = 0.196), and greater tongue volume (APDI: β = 0.174). Hyperdivergent vertical patterns—indicated by a lower ODI—were significantly correlated with a lower tongue tip position (β = –0.311) and posterior tongue position (β = –0.230). Regarding transverse dimensions, tongue volume showed positive correlations with upper intermolar width (β = 0.349), lower intercanine width (β = 0.130), lower intermolar width (β = 0.311), and a negative correlation with upper intercanine width (β = –0.299).

**Conclusions:**

Sagittal and vertical craniofacial patterns are interrelated and show associations with tongue and hyoid position, as well as tongue volume. Transverse dental arch dimensions are correlated not only with tongue position and volume but also with pharyngeal airway volume.

## Introduction

The position and function of the tongue has a significant association with the normal growth and development of dentofacial structures. Moss introduced the concept of the “functional cranial component,” referring to the skeletal and soft tissue components associated with functions such as respiration, speech, vision, and mastication [1]. He demonstrated that the growth of all skeletal structures is a secondary response to the primary morphogenetic demands of their respective soft tissues, including muscles, nerves, and the oral and nasal cavities, known as the “functional matrix.”

The effect of the tongue on maxillofacial structures begins early in embryogenesis. A study by Hong et al. showed that tongue development is associated with the growth of the anterior and posterior cranial base, the jaws, and nasopharyngeal structures [2]. Proffit et al. stated that the resting pressure of the tongue and cheeks, rather than the pressure exerted during swallowing and speech, primarily determines final tooth position [3]. Swinehart demonstrated that the expansive forces of the tongue result in the normal development of mandibular arch widths and concluded that the equilibrium of forces among the tongue, lips, and cheeks during growth usually maintains balance [4]. Cheng et al. observed that tongue pressure during swallowing is associated with vertical jaw dimensions [5].

It is widely accepted that a low tongue position, often linked to mouth breathing, is correlated with malocclusion. Earlier studies focused on the pharyngeal airway dimensions of mouth-breathing subjects, who typically exhibit a vertical growth pattern and narrow dental arches known as adenoid facies [6,7]. However, limited research has explored the tongue’s associations with different types of malocclusions. Most studies focused on Class III malocclusion and produced conflicting results, with some suggesting that anterior tongue position and macroglossia contribute to mandibular prognathism [8,9], while others found no correlation between tongue size and mandibular prognathism [10,11]. Additionally, most studies used two-dimensional (2D) lateral cephalograms, which only allow observation of the tongue dorsum. Studies using three-dimensional (3D) images analyzed a limited number of patients to understand various combinations of sagittal and vertical relationships.

Therefore, this study aimed to evaluate the relationship between tongue and hyoid position, tongue volume, pharyngeal airway dimensions, and varying craniofacial growth patterns in the sagittal, vertical, and transverse dimensions.

## Materials and methods

### Study subjects

This retrospective multi-center study was approved by the Institutional Review Board of Korea University Anam Hospital (2023AN0117) and the institutional review board of Korea National Institute for Bioethics Policy (P01-202302-01-045). Informed consent was waived by the Institutional Review Boards due to the retrospective nature of the study. All patients aged over 19 years who had undergone cone beam computed tomography (CBCT) for orthodontic diagnosis at Korea University Anam Hospital between March 2019 and February 2023 and at a private practice between January 2021 and January 2023 were included. Exclusion criteria included: (1) patients with a history of orthodontic treatment, (2) previous surgical interventions involving the tongue and palate, (3) respiratory or other systemic diseases, (4) craniofacial syndromes. The data from Korea University Anam Hospital and the private practice were accessed on May 1, 2023, and March 5, 2023, respectively, for this study. The data were anonymized before being obtained, so the authors did not have access to information that could identify individual participants during or after data collection.

### CBCT image acquisition

The subjects at Korea University underwent CBCT using the KaVo 3D eXam (Kavo, Biberach, Germany), with the following parameters: tube voltage, 90–120 kV; tube current, 3–8 mA; scan time, 24 s; voxel size, 0.20 mm; and field of view, 17 cm × 23 cm. The subjects at the private practice underwent CBCT using the Point 3D combi 500c (Pointnix, Seoul, Korea), with the following parameters: tube voltage, 50–90 kV; tube current, 4–10 mA; scan time, 19 s; voxel size, 0.183 mm; and field of view, 12 cm × 9 cm.

To standardize head orientation and enhance the reliability of CBCT imaging, patients were instructed to adopt a natural head position by looking at their reflection in a mirror. This posture was then maintained throughout the scanning process. The dentition was positioned in maximum intercuspation, with the lips and tongue in a relaxed state.

The CBCT data were converted and saved as digital imaging and communications in medicine (DICOM) files, which were then imported into the Invivo 6 (version 6.0, Anatomage, San Jose, CA) software for image visualization.

### Measurement of tongue position

**Fig 1:**
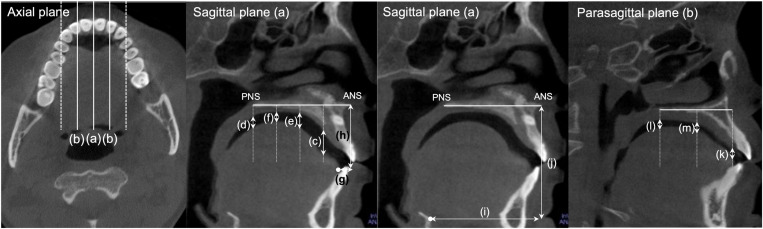
The plane perpendicular to the midsagittal reference plane (MSRP) and running through the anterior nasal spine (ANS) and posterior nasal spine (PNS) was defined as the palatal plane and used as a horizontal reference plane. Two additional planes parallel to the MSRP (a) and bisecting the line drawn from the MSRP to the most prominent points of the palatal cusps of the right and left first maxillary molars were constructed for tongue position measurements and defined as the lateral planes (b). Following variables were defined for the measurement of tongue and hyoid position in the MSRP: tongue mid 1 (c), the distance from the palate to the dorsum of the tongue at the posterior border of the incisive canal orifice; tongue mid 4 (d), the distance from the palate to the dorsum of the tongue along the line passing through the PNS; tongue mid 2 (e) and 3 (f), the distances from the palate to the dorsum of the tongue along the lines trisecting the reference lines for tongue mid 1 and 4; tongue tip x, horizontal distance from the ANS to the tongue tip (g); and tongue tip y, vertical distance from the horizontal reference plane to the tongue tip (h); hyoid x, horizontal distance from the ANS to the most superior anterior point of the hyoid (i); hyoid y, vertical distance from the horizontal reference plane to the most superior anterior point of the hyoid (j). Tongue positions were measured at three points in the lateral planes: at the most anterior portion of the tongue (k), at the PNS (l), and along the line bisecting the anterior and posterior points (m).

The CBCT images were reoriented using the midsagittal reference plane (MSRP), constructed by the anterior nasal spine (ANS), posterior nasal spine (PNS), and crista galli. The plane perpendicular to the MSRP and running through the ANS and PNS was defined as the palatal plane and used as a horizontal reference plane. Two additional planes parallel to the MSRP and bisecting the line drawn from the MSRP to the most prominent points of the palatal cusps of the right and left first maxillary molars were constructed for tongue position measurements. These were defined as the lateral planes (Fig 1). In each plane, the shortest distances from the dorsum of the tongue to the palatal gingiva were measured (Fig 1). The average of the right and left values denoted tongue position in the lateral plane.

### Tongue tip and hyoid positions

The horizontal and vertical positions of the anterosuperior-most point of the hyoid bone and tongue tip were measured on the MSRP. Linear distances from the palatal plane and a plane perpendicular to the palatal plane passing through the ANS were used as reference planes for measuring the vertical and horizontal positions of the tongue tip and hyoid, respectively (Fig 1).

### Tongue volume

**Fig 2:**
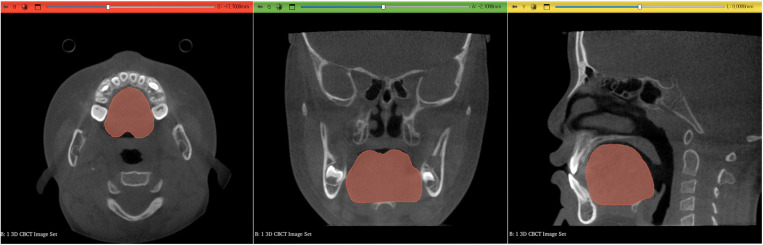
Tongue segmentation was done by defining the boundaries on axial, sagittal, and coronal image slices in 2D reformatted view. The lower border was the horizontal plane that passes the anterosuperior-most point of the hyoid bone.

Segmentation of the tongue was performed using 3D Slicer software [12]. 2D boundaries were interactively defined on axial, sagittal, and coronal image slices (Fig 2). The tongue segmentation was then cropped using horizontal and vertical planes intersecting the anterosuperior-most point of the hyoid bone. The 3D volume of the tongue segmentation was calculated in cubic centimeters.

### Measurement of pharyngeal airway

**Fig 3:**
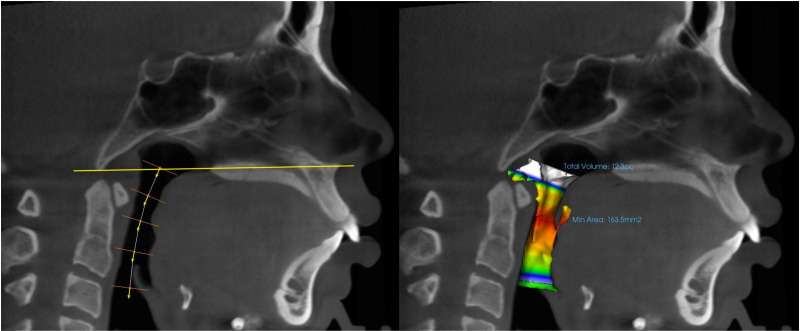
The superior and inferior borders were designated by drawing a line along the center of the pharyngeal airway in the sagittal section image, ensuring that the midpoint of the superior border crossed the palatal plane (ANS-PNS) and the inferior border crossed the base of the epiglottis.

The airway volume and minimum cross-sectional area were measured using the Airway module of the Invivo 6 software. The superior and inferior borders were designated using the specialized feature in the module by drawing a line along the center of the pharyngeal airway in the sagittal section image, ensuring that the midpoint of the superior border crossed the palatal plane (ANS-PNS) and the inferior border crossed the base of the epiglottis. The airway volume and minimal cross-sectional area were then automatically calculated (Fig 3).

### Lateral cephalometric analysis and dental arch dimensions

Lateral cephalometric images derived from CBCT scans were used for cephalometric analysis. After identifying the conventional hard-tissue cephalometric landmarks—sella (S), porion, basion, nasion (N), orbitale, point A (A), pogonion, gonion (Go), gnathion (Gn), point B (B), posterior nasal spine (PNS), anterior nasal spine (ANS), articulare, menton, maxillary incisor crown, maxillary incisor root, mandibular incisor crown, mandibular incisor root, maxillary first molar crown and root, mandibular first molar crown and root —were identified, and lateral cephalometric analysis was performed using the variables as below: SNA, SNB, ANB, anteroposterior dysplasia indicator (APDI), overbite depth indicator (ODI), body length, Frankfort-mandibular plane angle (FMA), mandibular plane angle (SN-GoGn), maxillary incisor inclination (U1-FH), incisor mandibular plane angle (IMPA), interincisal angle (IIA), overjet, and overbite. Transverse dimensions of the upper and lower dental arches were measured directly from the CBCT scans: upper intercanine width (U3C) and lower intercanine width (L3C) measured at the cusp tips, and upper intermolar width (U6F) measured at the root furcation and lower intermolar width (L6BC) measured at the mesiobuccal cusp tip. All landmark identifications and measurements were performed by the same investigator (JHK).

### Error of measurement

To assess intraexaminer reliability, tongue and airway measurements were repeated by the same investigator in 50 randomly selected patients two weeks after the initial evaluation. The intraclass correlation coefficients (ICCs) ranged from 0.92 to 0.98, indicating excellent reliability. Interexaminer reliability was evaluated in a separate set of 50 randomly selected patients, with all measurements performed by a second investigator (NJ). The resulting ICCs ranged from 0.82 to 0.93, also reflecting excellent agreement. Method error was calculated using Dahlberg’s formula, yielding errors ranging from 0.21 to 0.62 mm for linear measurements and 0.17° to 0.73° for angular measurements. Errors for tongue volume and airway volume were 0.35 cm³ and 0.22 cm³, respectively.

### Statistical analysis

Given the close correlations observed among cephalometric variables, multivariate linear regression analysis was employed to evaluate the associations between tongue and hyoid positions, tongue volume, and pharyngeal airway dimensions with sagittal and vertical skeletal patterns and transverse dental arch dimensions. This statistical method allows for the assessment of linear relationships between multiple independent and dependent variables. Two multivariate linear regression models were developed. The first model employed ANB, APDI, ODI, and SN-GoGn as outcome variables to represent participants’ sagittal and vertical craniofacial patterns. The second model utilized upper intercanine width (U3C), upper intermolar width (U6F), lower intercanine width (L3C), and lower intermolar width (L6BC) as outcome variables. In both models, variables related to tongue and hyoid position, tongue volume, and airway dimensions served as predictor variables.

Furthermore, canonical correlation analysis was performed to evaluate potential associations among different predictor variable groups:

Cluster 1 (C1): Hyoid variable cluster, consisting of hyoid x and hyoid y.

Cluster 2 (C2): Tongue variable cluster, consisting of tongue tip x and y, as well as tongue mid 1, 2, 3, and 4.

Cluster 3 (C3): Pharyngeal airway variable cluster, consisting of airway volume and minimal cross-sectional area.

All statistical calculations were performed using IBM SPSS Statistics for Windows (Version 20.0; IBM Corp., Armonk, NY, USA). Statistical significance was set at *P* < 0.05.

## Results

### Descriptive statistics

A total of 185 non-growing subjects (72 males and 113 females; mean age, 28.7 ± 9.5 years) were included in the study. The lateral cephalometric analysis, hyoid and tongue positions, tongue volume and airway volume, and dental arch widths of the subjects are shown in Table 1. The subjects had a mean ANB of 3.5 ± 2.6, and mean SN-GoGn of 37.0 ± 6.2 (Table 1). When classified by sagittal skeletal pattern, 54% were classified as Class I (0 < ANB < 4), 38% as Class II (ANB ≥ 4), and 8% as Class III (ANB ≤ 0). When classified by vertical skeletal pattern, 17% were hypodivergent (SN-GoGn ≤ 31), 46% were normodivergent (31 < SN-GoGn < 39), and 37% were hyperdivergent (SN-GoGn ≥ 39) growth patterns.

**Table 1:** Lateral cephalometric, hyoid, tongue, airway and dental analysis of the subjects (n = 185).

Variables	Mean	Standard deviation
ANB (°)	3.5	2.6
APDI (°)	82.0	6.4
ODI (°)	71.6	6.6
SN-GoGn (°)	37.0	6.2
Hyoid x (mm)	54.8	6.0
Hyoid y (mm)	58.8	6.0
Tongue tip x (mm)	3.4	3.6
Tongue tip y (mm)	23.9	6.4
Tongue mid 1 (mm)	2.8	4.2
Tongue mid 2 (mm)	2.8	3.8
Tongue mid 3 (mm)	2.7	3.7
Tongue mid 4 (mm)	3.1	4.1
Tongue volume (cm^3^)	69.1	10.4
Airway volume (cm^3^)	15.3	7.5
Airway min CSA (mm^2^)	146.6	75.9
U3C (mm)	43.4	5.7
U6F (mm)	40.7	7.1
L3C (mm)	27.8	2.7
L6BC (mm)	51.2	6.6

ANB, angle formed by the nasion, point A, and point B; APDI, anteroposterior dysplasia indicator; ODI, overbite depth indicator; SN-GoGn, the angle between the SN plane and Go-Gn line; Hyoid x, horizontal distance from the most superior point of the hyoid bone to the ANS; Hyoid y, vertical distance from the most superior point of the hyoid bone to the ANS; tongue tip x, horizontal distance from the most superior point of the tongue tip to ANS; tongue tip y, vertical distance from the most superior point of the tongue tip to ANS; tongue mid 1: vertical distance from the incisive canal orifice to the dorsal side of the tongue; tongue mid 2: divide the distance between tongue mid 1 and tongue mid 4 into thirds, the vertical distance from the 1/3rd point to the dorsal side of the tongue; tongue mid 3: divide the distance between tongue mid 1 and tongue mid 4 into thirds, the vertical distance from the 2/3rd point to the dorsal side of the tongue; tongue mid 4: vertical distance from the PNS to the dorsum of the tongue; min CSA, minimum cross-sectional area of the airway; U3C, upper intercanine width measured at the cusp tip; U6F, upper intermolar width measured at the root furcation, L3C, lower intercanine width measured at the cusp tip; L6 BC, lower intermolar width measured at the mesiobuccal cusp tip.

### Multivariate linear regression analysis

Multivariate linear regression analysis for sagittal and vertical skeletal patterns, represented by ANB, APDI, ODI, and SN-GoGn, showed significant associations with the following parameters: hyoid x, tongue tip y, tongue volume, and tongue mid 4 (Table 2). For transverse dental arch widths, which used U3C, U6F, L3C, and L6BC as outcome variables, significant associations were observed for tongue volume, tongue tip y, and airway volume (Table 3). The effects of age and sex on the outcome variables did not show statistical significance and thus were excluded from the regression analysis. The estimated regression coefficients for the outcome variables are shown in Tables 4 and 5.

**Table 2:** Multivariate linear regression analysis for sagittal and vertical skeletal patterns, represented by point A-Nasion-point B(ANB), anteroposterior dysplasia indicator (APDI), overbite depth indicator (ODI) and SN to mandibular plane angle (SN-GoGn), given the tongue and hyoid positions, tongue volume and pharyngeal airway dimensions.

Predictor variable	Wilks’ Lambda	F	*P* value	Observed Power
Hyoid x	.872	6.2	<.001	.987
Tongue tip y	.897	4.8	.001	.953
Tongue volume	.942	2.6	.037	.723
Tongue mid 4	.927	3.3	.012	.835

Hyoid x, horizontal distance from the most superior point of the hyoid bone to the line perpendicular to the palatal plane and crossing anterior nasal spine; tongue tip y, vertical distance from the most superior point of the tongue tip to the palatal plane; tongue mid 4: vertical distance from the posterior nasal spine to the dorsum of the tongue.

**Table 3:** Multivariate linear regression analysis for transverse dental arch widths, represented by upper intercanine width (U3C), upper intermolar width (U6F), lower intercanine width (L3C) and lower intermolar width (L6BC), given the tongue and hyoid positions, tongue volume and pharyngeal airway dimensions.

Predictor variable	Wilks’ Lambda	F	*P* value	Observed Power
Tongue volume	.718	1.6	<.001	1.000
Tongue tip y	.845	5.0	.001	.955
Airway volume	.877	3.8	.007	.878

Tongue tip y, vertical distance from the most superior point of the tongue tip to the palatal plane.

**Table 4:** Estimated regression coefficients for the sagittal and vertical skeletal patterns.

Outcome variable	Predictor variable	Beta	*P* value
ANB	Hyoid x	.249	.001
	Tongue tip y	−.231	.002
	Tongue volume	−.053	.473
	Tongue mid 4	−.186	.010
APDI	Hyoid x	−.291	<.001
	Tongue tip y	.166	.021
	Tongue volume	.174	.018
	Tongue mid 4	.196	.006
ODI	Hyoid x	−.013	.864
	Tongue tip y	−.311	<.001
	Tongue volume	.006	.940
	Tongue mid 4	−.230	.002
SN-GoGn	Hyoid x	.099	.208
	Tongue tip y	.106	.170
	Tongue volume	−.091	.252
	Tongue mid 4	.007	.932

**Table 5:** Estimated regression coefficients for the transverse dimensions.

Outcome variable	Predictor variable	Beta	*P* value
U3C	Tongue volume	−.299	.017
	Tongue tip y	−.211	.001
	Airway volume	.204	.021
U6F	Tongue volume	.349	<0.001
	Tongue tip y	.433	<0.001
	Airway volume	−.100	.209
L3C	Tongue volume	.130	<0.001
	Tongue tip y	.366	.140
	Airway volume	−.113	.201
L6BC	Tongue volume	.311	<0.001
	Tongue tip y	.458	<0.001
	Airway volume	−.183	.019

U3C, upper intercanine width measured at the cusp tip; U6F, upper intermolar width measured at the root furcation, L3C, lower intercanine width measured at the cusp tip; L6BC, lower intermolar width measured at the mesiobuccal cusp tip; tongue tip y, vertical distance from the most superior point of the tongue tip to the palatal plane.

### Sagittal and vertical skeletal patterns

According to the regression coefficients, increased APDI and decreased ANB, indicating a skeletal Class III tendency, showed significant correlations with anteriorly positioned hyoids (*P = *0.001 and *P *< 0.001 for ANB and APDI, respectively, Table 4) and lower tongue positions at the tongue tip (*P = *0.002 and 0.021 for ANB and APDI, respectively, Table 4) and in the posterior area (*P = *0.010 and *P = *0.006 for ANB and APDI, respectively, Table 4); tongue volume showed a positive correlation with APDI (*P = *0.018), but no significant correlation was observed with ANB (*P = *0.473, Table 4).

A decrease in ODI (indicating a hyperdivergent growth pattern) showed a significant correlation with lower tongue positions at the tongue tip and in the posterior area (*P* *< *0.001 and *P = *0.002 for tongue tip y and tongue mid 4, respectively, Table 4). SN-GoGn showed a similar pattern of correlation with the tongue position but lacked statistical significance (*P* *< *0.05, Table 4).

ANB, angle between the nasion-point A line and nasion-point B line; APDI, anteroposterior dysplasia indicator; ODI, overbite depth indicator; SN-GoGn, the angle between the SN plane and Go-Gn line; hyoid x, horizontal distance from the most superior point of the hyoid bone to the line perpendicular to the palatal plane and crossing anterior nasal spine; tongue tip y, vertical distance from the most superior point of the tongue tip to the palatal plane; tongue mid 4: vertical distance from the posterior nasal spine to the dorsum of the tongue.

### Dental arch widths

The width of the dental arch showed a correlation with tongue volume, tongue position, and airway volume. Tongue volume had a positive correlation with U6F, L3C, and L6BC (*P* *< *0.001, Table 5), but a negative correlation with U3C (*P = *0.017, Table 5). An inferior position of the tongue tip was correlated with decreased U3C (*P = *0.001, Table 5) and increased U6F and L6BC (*P* *< *0.001, Table 5). Airway volume was positively correlated with U3C and negatively correlated with L6BC (*P = *0.021 and 0.019 for U3C and L6BC, respectively, Table 5).

### Canonical correlation analysis

A significant correlation was observed between the hyoid position variable group (C1) and the tongue position variable group (C2) with a correlation coefficient of 0.366 (*P *< 0.001, Table 6). However, the correlations between the hyoid position variable group (C1) and the airway dimensions variable group (C3), and between the tongue position variable group (C2) and the airway dimensions variable group (C3), were not statistically significant (*P *> 0.05, Table 6).

**Table 6:** The first canonical correlation coefficient among the clusters of variables related to hyoid (C1: hyoid x, hyoid y), tongue (C2: tongue tip x, tongue tip y, tongue mid 1, tongue mid 2, tongue mid 3, tongue mid 4), and airway (C3: total airway volume, min CSA).

Groups	Coefficient	P value
C1-C2	0.366	<0.001
C1-C3	0.269	0.060
C2-C3	0.276	0.469

Hyoid x, horizontal distance from the most superior point of the hyoid bone to the ANS; Hyoid y, vertical distance from the most superior point of the hyoid bone to the ANS; tongue tip x, horizontal distance from the most superior point of the tongue tip to ANS; tongue tip y, vertical distance from the most superior point of the tongue tip to ANS; tongue mid 1: vertical distance from the incisive canal orifice to the dorsal side of the tongue; tongue mid 2: divide the distance between tongue mid 1 and tongue mid 4 into thirds, the vertical distance from the 1/3rd point to the dorsal side of the tongue; tongue mid 3: divide the distance between tongue mid 1 and tongue mid 4 into thirds, the vertical distance from the 2/3rd point to the dorsal side of the tongue; tongue mid 4: vertical distance from the PNS to the dorsum of the tongue; min CSA, minimum cross-sectional area of the airway

## Discussions

The role of normal oral and nasopharyngeal functions, such as breathing, chewing, and swallowing, and their associations with adjacent structures have been recognized since the early stages of growth and development. Given the high individual variation in pharyngeal airway and tongue position and volume, this multi-center retrospective study was conducted to investigate how the soft tissue components of the oropharyngeal “functional matrices” are associated with dentoskeletal growth. The study utilized a large sample size to account for this variability and included a range of different dental and skeletal patterns. Despite the high variability of tongue and airway measurements, it has been reported that these measurements are reliable when taken in the natural head position [13].

Tongue position measured in the lateral part of the tongue dorsum showed inconsistent values and no significant correlations with dentoskeletal patterns in our subjects. Anatomically, the lateral part of the tongue is positioned closer to the palate (Fig 2). Consequently, the distance between the palate and the lateral part of the tongue exhibited minimal variation, frequently showing direct contact. This inherent anatomical characteristic likely accounts for the lack of significance of the lateral tongue position as predictor variables. However, tongue position measured in the midsagittal plane, including the tongue tip, showed significant associations with the dentoskeletal patterns of the subjects.

Since the muscles of the tongue originate from the hyoid, a significant correlation between tongue position and hyoid position was observed in the canonical correlation analysis. Nevertheless, the function of the hyoid bone extends beyond providing an anchor point for tongue and pharyngeal muscles; it also provides longitudinal traction to tracheal, laryngeal, and upper esophageal structures [14]. Consequently, the range of hyoid position was limited and showed less association with dentoskeletal patterns than tongue measurements.

Our sample reflects the distribution of patients in real-world clinical settings. Although Class I and normodivergent patterns predominated, our dataset still included a broad spectrum of skeletal characteristics. Therefore, to account for variability and minimize the impact of class imbalance, cephalometric variables were analyzed as continuous measures—using ANB and APDI instead of Angle’s classifications (Class I, II, III) for sagittal skeletal patterns, and ODI and SN-MP instead of classifications of hyperdivergent, normodivergent, and hypodivergent for vertical skeletal patterns. Additionally, since sagittal and vertical skeletal patterns are not independent features and showed significant correlations with each other in our subjects, they were included as multiple outcome variables in a single regression model. This approach considers possible interaction effects and their joint association with predictors. However, transverse dimensions, represented by intercanine and intermolar widths in the upper and lower arches, were independent of sagittal and vertical patterns, showing low correlations with them, and thus were analyzed separately.

Regarding predictor variables, the results indicated that while sagittal and vertical patterns shared common predictors, transverse patterns showed associations with a different set of predictor variables. For sagittal and vertical skeletal patterns, vertical tongue position, tongue volume, and hyoid sagittal position were significantly associated. Our results indicated that mandibular prognathism was correlated with higher tongue volume and lower tongue position. Similarly, Primozic et al. reported low tongue position in the posterior and middle of the tongue and higher mouth floor volume in Class III subjects [15]. Our results for tongue volume were also consistent with those reported by Ihan Hren and Barbic [9], and Iwasaki et al. [16], but contrasted to those of Yoo et al [11]. Yoo et al. compared tongue volume between individuals with normal occlusion and those with mandibular prognathism, finding no statistically significant difference. However, despite the lack of group differences, they reported that lateral cephalometric variables—such as facial angle, facial convexity angle, and Y-axis angle, which represent sagittal and vertical skeletal patterns—showed significant correlations with tongue volume.

Excessive vertical growth patterns were correlated with a low tongue position measured in the posterior area and tip of the tongue. Unlike the sagittal pattern, tongue volume showed no correlation with vertical skeletal patterns. Our findings on tongue position and vertical patterns align with previous studies showing significantly lower tongue positions in hyperdivergent patients compared to normo- and hypodivergent patients [17,18]. One possible explanation is that hyperdivergent patients have long and narrow airways and tend to mouth breathe to compensate for the narrow airway, leading to lower tongue posture to maintain the oral airway [19,20]. However, pharyngeal airway volume did not show a significant association with vertical skeletal patterns.

Transverse dimensions showed correlations with the vertical position of the tongue tip, tongue volume, and pharyngeal airway volume. Interestingly, the correlation pattern between the tongue tip and upper intercanine width was different from that observed with upper intermolar, lower intercanine, and lower intermolar widths. As the tongue tip was positioned inferiorly, the upper intercanine width tended to decrease, while the upper intermolar, lower intercanine, and lower intermolar widths increased. This may be due to the shape of the tongue in the transverse dimension, where lower tongue position leads to lateral expansion of the tongue body, resulting in the observed pattern of transverse dimensions. A similar pattern was observed for tongue volume, where increased tongue volume was correlated with increased dimensions in the upper intermolar, lower intercanine, and lower intermolar widths. This could be further explained by the positive correlation between tongue volume and lower tongue position in the anterior and middle part of the tongue. Primozic analyzed the cross-sectional analysis of the maxilla and the mandible, showing a significant correlation with posterior tongue position. Tariq et al. studied the relationship between tongue position and lower dental arch widths, finding a correlation between mandibular arch width and tongue posture [18]. Pitts et al. studied tongue force in the anterior and posterior parts and palatal dimensions, reporting that increased tongue volume was associated with increased posterior tongue strength, and this was associated with increased palatal width in the upper molars [21].

Pharyngeal airway volume was positively correlated with upper intercanine width and negatively correlated with lower intermolar width. This pattern might be associated with tongue posture, as those with decreased airway dimensions have a lower tongue position and narrow upper arch, while the lower arch width increases due to the low tongue position. Rana et al. found a significant negative correlation between tongue volume and oropharyngeal airway volume [22]. Zeng et al. studied the relationship between tongue position and volume, reporting that patients with an extremely low tongue position showed decreased upper airway volume. Another notion is that the pharyngeal airway is surrounded by soft tissues, including the tongue. Therefore, an increase in tongue volume, such as in obese patients [23,24], may enlarge the tongue anteroposteriorly, resulting in decreased upper airway volume and caudal displacement of the hyoid [25,26].

While sagittal, vertical skeletal patterns, and transverse patterns showed significant correlations with tongue position, volume, and airway volume, incisor patterns showed limited correlation with predictor variables. Variables related to incisor position, such as overbite, overjet, IMPA, U1-FH, and IIA, demonstrated no significant correlation in the multivariate regression analysis. However, overbite showed a significant correlation in the univariate correlation analysis, indicating a tendency for shallow overbite in cases with an anterior tongue position. These findings suggest that further investigation focusing on dental measurements and factors associated with dental patterns is warranted.

Our findings suggest that factors associated with tongue posture, parafunctional habits, and breathing patterns, may be correlated with variations in facial skeletal and dental patterns. Therefore, early myofunctional therapy interventions addressing aberrant tongue posture and breathing patterns in growing children may be considered as a supportive approach for facilitating optimal growth. However, it is crucial to acknowledge that this study design does not allow for definitive conclusions regarding causality. Notably, a complex interaction exists between genetics and functional factors in craniofacial growth, and it is plausible that the observed tongue and airway characteristics are adaptations to a genetically predetermined skeletal pattern.

One limitation of this study is the use of dental arch widths as the sole outcome measures for the transverse analysis. Although dental arches are in direct contact with the tongue and airway, incorporating skeletal transverse dimensions of the maxilla and mandible could offer additional insight into the complex interplay among the tongue, hyoid, and airway in craniofacial development. Another limitation arises from the retrospective design of the study, which may have introduced variation in head posture. While CBCT scans were acquired using a standardized protocol in natural head position, subtle variations could have affected tongue and airway measurements. Additionally, potential errors in tongue segmentation may have affected the results, as the process was performed manually. Lastly, as this was a multi-center study, two different CBCT machines were used. Variations in imaging protocols between centers may have also contributed to measurement discrepancies.

## Conclusions

The results of this study indicate that sagittal and vertical craniofacial patterns are interrelated and demonstrate correlations with tongue and hyoid position, as well as tongue volume. Transverse dental arch dimensions are correlated not only with tongue position and tongue volume, but also with pharyngeal airway volume. Contrastingly, the correlation between incisor patterns and the tongue, hyoid, and pharyngeal airway appears limited.
